# The Institutional Library

**Published:** 1913-09-13

**Authors:** 


### THE INSTITUTIONAL LIBRARY.

### The Creator of the Modern Hospital.

Lord Lister : His Life and Work. By G. T. Wrench, M.D.(Lond.). (T. Fisher Unwin, Adelphi Terrace. Price 15s. net.)

The ordinary hospital man, whether medical or layman, takes the fact that Lister is the creator of the modern hospital so much for granted that he does not pause to understand what it means. That is why the medical student, the hospital manager, and the resident medical officer should read this book. It will tell him frankly and terribly, in the appalling but necessary third chapter, which becomes a document from its full quotations from John Bell's discourses, what hospitals were like before the days of antisepsis. Hospitals in those days were places for studying the phenomena of suppuration, and, virtually, nothing else; but pyæmia and hospital gangrene, which rotted the tissues with a virulence no wild beast could surpass, were not studied, they were accepted; indeed, they were admired. John Bell's discourses remain to show how far this statement falls short of exaggeration. The humour of Sir James Simpson's retort on the clergy, who denounced anæsthesia as a sacrilegious interference with the Divine dispensation of pain, that the first recorded operation in the world, the removal of Adam's rib, was performed under an anæsthetic since God caused a deep sleep to fall upon Adam, must not hide from us the appalling conditions in hospitals, and the indifference which accompanied them. If ever a remark were inspired, it was this of Sir James Simpson's, for profundity and humour are the two distinguishing qualities of the deepest truth. With such a great subject to deal with in popular fashion, for Dr. Wrench's work has the merit of that intention even if the publisher has done his best to defeat it by the absurd price of 15s., it is not surprising that the style of the author at first suffers from exuberant excrescences; indeed he falls into downright nonsense at times. Thus, Lister begins by being the ordinary stuffed hero of all the bad biographies, and on page 44 the statement that Lister "was fond of music" is followed, actually in the next sentence, by the remark that "he probably never went of his own free will to a concert in his life." Dr. Wrench cannot have it both ways. Chapter VIII. stylistically is the worst, but after that the author becomes infected by the surpassing interest of his subject, and to the end there is nothing more to complain of in this respect. If hospitals had been run by a trust to boom the coffin trade they could not have had more need of them, and the overcrowding of industrial centres was positively intensified by the presence of a hospital. The remedy was worse than the disease—a fact worth the humble attention of all hospital men to-day, when we remember that, if 1879 may be taken as the year in which Lister's principles were first practised on at all an adequate scale nationally, how few medical men or managers can have much memory of the preceding putrescence of the wards. Again, we must repeat, such words are the reverse of exaggeration. Suppuration was declared inevitable so long as ligatures were inevitable. Another point of vital interest in this book is the examples which it gives of the way in which pioneers oppose each other. The inventor of chloroform opposed the perfecter of antisepsis, and Syme (Lister's father-in-law, of all people!) denounced the former's pamphlet on acupressure and ligatures as "vulgar insolence." Then comes the fascinating history of absorbable ligatures. Of even greater interest to institutional workers is, however, the effect of antisepsis on hospital construction. Despair had become so great that Simpson, in his characteristically hasty and drastic way, declared that all hospitals should be destroyed, so great was the difference between the fatalities that occurred in hospitals, and those which were avoided even in the houses of the poor. Lister's famous paper on "The Salubrity of Hospitals" remains to show that hospitals were frequently built on cesspools or burial-grounds, as in the case of the Glasgow Infirmary, and also that where antisepsis was rigidly practised such conditions were by no means fatal to success. In Lister's hands it secured results of which the modern hospital might still be proud. The question then arises : Is the immense expenditure on hospital construction of late years really justified? It is a searching question which the modern hospital must face. Many millions of pounds have to justify themselves. Can they do it? We very much doubt if they can. In a very provocative chapter, which every hospital man should make a point of reading, an attack is made on the aseptic system, which is denounced as a retrogression from Lister's methods; and its elaboration is contrasted unfavourably, of course, with Lister's simplicity. In one of the numerous quotations from medical men, with which Dr. Wrench embellishes his criticisms, on page 326 we read : "Never before have architects been more indispensable. The least defect in the condition of an operation room manifests itself by disasters," and so forth. The whole of this chapter—XIII.—will be adversely received, but that is no reason for skipping it. Want of space forbids any further detailed treatment of this book, which, though in no sense a great biography, has this outstanding merit, that the author, in the later chapters, has caught the philosophic outlook on medicine which was Lister's distinguishing mark—an infection which is really the greatest tribute to Lister in the book. We heartily endorse his plea for the issue of a cheap edition of Lister's Works, and suggest that the Clarendon Press should supplement their Library Edition of 1909 with a useful and much-needed reprint of this character.

### Institutional Treatment of Tuberculosis.

The State Provision of Sanatoriums. By S. V. Pearson, M.D.(Cantab.), M.R.C.P.(Lond.). (Cambridge University Press. 3s. net.)

This little volume makes an opportune appearance when so many authorities are devoting their attention to the provision of institutions for the treatment of tuberculosis. It is written especially for "that numerous band of interested and disinterested workers amongst county councillors, doctors, and National Health Insurance Committeemen, in whose hands the management of State sanatoriums lies." It is to be hoped that it will find its way at any rate into the hands of the lay members of the various committees, and that they will act upon the advice therein given. At the present moment few of the laity fully realise the extent of the problem which confronts the public health authorities, but this little book should help them to understand and appreciate it.

Dr. Pearson is apparently not convinced that tuberculosis dispensaries are of much value except as information bureaus and centres of instruction, but until a far larger number of beds is available in institutions, it is difficult to see how otherwise to provide any treatment for a large proportion of patients—a difficulty which will certainly be accentuated when the dependants of insured persons become entitled to benefit under the Insurance Act. The author appreciates the difficulty and cost of providing institutional treatment, and devotes a chapter to the consideration of the various possible methods of raising communal funds for the provision and maintenance of sanatoriums.

The principles of construction and architectural arrangements are dealt with clearly and succinctly, and the ideas here set forth should be of use to architects whose experience in this class of work is limited. Some plans for a building to contain 250 beds are included at the end of the volume, and demonstrate the principles now generally accepted in the construction of sanatoriums. Dr. Pearson very rightly insists on the necessity of having an adequate staff in these institutions, where the personal attention required from the doctor is considerable, and strongly deprecates any continuance of the present hospital system under which the junior staff practically give their services. He considers that the remuneration should be adequate and direct, so that experience, advertisement, and honour shall not take the place of those monetary rewards to which the staff are entitled. The advantages of sanatorium treatment over domiciliary are discussed both from a curative and an educational standpoint. The question as to whether the hospitals or the sanatoriums are to be the teaching centres for future medical students in this special subject is considered, and some arguments for and against are mentioned. A short bibliography is included. The general appearance and printing of the book are excellent, and it can be thoroughly recommended to all those interested in the institutional treatment of tuberculosis.

### Sir Horace Plunkett on Modern Medicine.

Some Tendencies of Modern Medicine from a Lay Point of View. By the Right Hon. Sir Horace Plunkett, K.C.V.O., F.R.S. (Dublin: Eason and Son, Limited. Price 6d.)

It has long been recognised by wise men that the layman is the most suggestive critic of the learned professions, which, outside their own technics, are apt to be blind. Sir Horace Plunkett is, therefore, following the most respectable precedent in discussing some tendencies of modern medicine, which the hospital world is too much engrossed with to orientate as a whole. After stating his opinion that the existing relation between the public and the medical profession is not such as to engender mutual confidence and co-operation on the most productive scale, he proceeds to contrast his own, doubtless also the general layman's, experience of private practice with the system which he found on visiting a patient at the Battle Creek Sanitarium (the spelling is justified in an amusing footnote), Michigan, U.S.A. Although the successors of the Founders, who were Seventh Day Adventists, still keep Sunday on Saturday, Sir Horace Plunkett devotes some fifteen out of his thirty-one pages to showing that the sanitarium is not the freak of faddists, nor, indeed, connected otherwise with Adventists at all. Dr. J. H. Kellog, who is the medical superintendent, receives high praise for the system which he controls, which may be inadequately summarised as follows: Based on scientific medical philanthropy, like a voluntary hospital, the sanitarium, which "has the same recognition by the Edinburgh Examining Board as any other American hospital," aims at systematic diagnosis by elaborate institutional means, somewhat analogous to those employed at the Mayo Hospital; and at making available every rational curative method, while giving prominence to "physical therapy or so-called physiologic therapeutics." It also attempts to combine the technical advantages of a hospital with the comforts of a modern hotel, and to carry on research, especially in relation to diet and nutrition. The writer expresses himself completely satisfied that philanthropy and not commercial gain is its basis; describes the work of its medical missionaries; the elaborate diagnosis which his own case received (he went there as a visitor, but became a patient during his stay); gives the "unconventional prescription" with which he was furnished; urges the importance of educating the patient in his condition for the sake of both patient and the profession; and, finally, attacks the question of dietetics, inclining to a modified vegetarianism. His constructive suggestion is "a co-ordination of existing clinics with a definite assignment to each of a certain part of the diagnostic work, and an agreement among doctors to use the several institutions in private practice." We have preferred to quote and summarise rather than to criticise and debate. The Hospital reader will thus realise that not merely the efficiency of the present system has to be considered, but also whether there may not be a different system which is better, at any rate as regards private practice. It is not only the lay public, we hope, which has caused this pamphlet to run into a second impression.

The Laws of Sexual Philosophy. By J. L. Chundra, L.M.S. (Calcutta. 1913.)

The scientific study of the subject to which this book is devoted is undoubtedly to be encouraged, and plain speech on this matter is highly desirable. This book, however, is a tissue of absurdities from start to finish; its perusal can do no good to anybody, and may easily do harm to many. It is difficult to decide whether the ignorance, the conceit, or the superstition of the author is the greatest.

### Canons of Military Surgery.

Surgical Experiences in South Africa. By G. H. Makins, C.B., F.R.C.S. Second Edition. (London: Oxford University Press. 1913. Pp. 504. Price 10s. 6d. net.)

In a prefatory note the author explains that this book, though long out of print, is constantly asked for whenever a war breaks out. So he has issued a second edition, more than twelve years after the first one, merely bringing up to date some of the case histories which have been traced further during the interval. And it is easy to see why this recurring demand for the book takes place; for Mr. Makins' conclusions as to the correct surgical principles for dealing with modern bullet wounds have stood the test of time exceedingly well. Indeed, we may say that they have passed into accepted canons of military surgery; and probably many who believe in them implicitly are quite unaware who it was that first formulated them. It is getting rather a far cry now to Magersfontein and Modder River—nearly fourteen years—and a book based on the combatant aspects of the South African War might well prove tedious. Mr. Makins sticks closely to his last, however, and his book has, in consequence, a more enduring and permanent kind of popularity among military surgeons than a less technical account of war experiences could attain. Excellently produced, we do not doubt that the demand for this second edition will continue to revive, whenever wars and rumours of wars arise, for many years.

